# Harmonization Process and Reliability Assessment of Anthropometric Measurements in the Elderly EXERNET Multi-Centre Study

**DOI:** 10.1371/journal.pone.0041752

**Published:** 2012-07-31

**Authors:** Alba Gómez-Cabello, Germán Vicente-Rodríguez, Ulrike Albers, Esmeralda Mata, Jose A. Rodriguez-Marroyo, Pedro R. Olivares, Narcis Gusi, Gerardo Villa, Susana Aznar, Marcela Gonzalez-Gross, Jose A. Casajús, Ignacio Ara

**Affiliations:** 1 GENUD (Growth, Exercise, NUtrition and Development) Research Group, University of Zaragoza, Zaragoza, Spain; 2 Faculty of Health and Sport Science (FCSD), Department of Physiatry and Nursing, University of Zaragoza, Huesca, Spain; 3 ImFINE Research Group, Department of Health and Human Performance, Technical University of Madrid, Madrid, Spain; 4 GENUD (Growth, Exercise, NUtrition and Development) Toledo Research Group, University of Castilla-La Mancha, Toledo, Spain; 5 Institute of Biomedicine (IBIOMED), University of León, León, Spain; 6 Faculty of Sport Sciences, University of Extremadura, Cáceres, Spain; 7 PAFS (Promoting Physical Activity for Health) Research Group, University of Castilla La Mancha, Toledo, Spain; University of Zaragoza, Spain

## Abstract

**Background:**

The elderly EXERNET multi-centre study aims to collect normative anthropometric data for old functionally independent adults living in Spain.

**Purpose:**

To describe the standardization process and reliability of the anthropometric measurements carried out in the pilot study and during the final workshop, examining both intra- and inter-rater errors for measurements.

**Materials and Methods:**

A total of 98 elderly from five different regions participated in the intra-rater error assessment, and 10 different seniors living in the city of Toledo (Spain) participated in the inter-rater assessment. We examined both intra- and inter-rater errors for heights and circumferences.

**Results:**

For height, intra-rater technical errors of measurement (TEMs) were smaller than 0.25 cm. For circumferences and knee height, TEMs were smaller than 1 cm, except for waist circumference in the city of Cáceres. Reliability for heights and circumferences was greater than 98% in all cases. Inter-rater TEMs were 0.61 cm for height, 0.75 cm for knee-height and ranged between 2.70 and 3.09 cm for the circumferences measured. Inter-rater reliabilities for anthropometric measurements were always higher than 90%.

**Conclusion:**

The harmonization process, including the workshop and pilot study, guarantee the quality of the anthropometric measurements in the elderly EXERNET multi-centre study. High reliability and low TEM may be expected when assessing anthropometry in elderly population.

## Introduction

Aging is accompanied by an increase of body weight and fat mass [1], [2], being of great importance due to the fact that both are independent risk factors for chronic diseases in elderly people [3]. This has created a need for accurate assessment of body composition and fat distribution in epidemiological studies aiming to study the interaction of behavioral, environmental and genetic indices in the development and progression of chronic diseases. There are several accurate methods for assessing body composition, like dual X-ray absorptiometry (DXA), underwater weighing, air displacement plethysmography, computer tomography and nuclear magnetic resonance [4]–[6]. However, these techniques are expensive and require sophisticated laboratory settings, which make them a challenge to be used in large epidemiological studies. However, anthropometry is a portable, non-invasive, inexpensive, and useful method in field studies and for these reasons, researchers have considered it as feasible method to use in large cohorts.

The precision of anthropometric measurements plays an important role in delivering meaningful information for the subjects’ nutritional status [6]. As with any use of quantitative biological measures, it is important to minimize error, and to know and understand the various ways in which it is estimated and assessed [7]. Reliability is the degree to which within-subject variability is due to factors other than measurement error. The lower the variability between repeated measurements of the same subjects by one (intra-rater differences) or two or more observers (inter-rater differences), the greater the precision. Determination of intra- and inter-rater variability is important in improving measurement precision and reliability [8]. The most common measurements of precision are the technical error of measurement (TEM) and the coefficient of reliability (*R*). The use of two error estimates, TEM and *R*, can provide most of the information needed to determine whether a series of anthropometric measurements can be considered accurate. Unreliable measurement of the exposure variable can dilute or attenuate the observed association of the variable with the disease of interest, thereby reducing the power of the study to detect a true association [7]. The elderly EXERNET multi-centre study aimed to describe the total body fat percentage and anthropometric indices of body fat distribution of Spanish elderly from anthropometrics [9]. As in large size cohorts in whom data collection is performed by several researchers, the chances for systematic and random errors increase; therefore, it was decided before the implementation of the elderly EXERNET multi-centre study to proceed with the harmonization of anthropometric measurements as an essential factor to ensure high reliability measurements among all observers participating in the study. Moreover, due to the changes of body tissues that constantly take place during life, it would be important to elucidate if high reliabilities of anthropometrics can be also achieved in elderly people. Due to their great importance in terms of health; height [10], knee height [11], waist and hip circumferences [12] were the anthropometric measurements selected in the evaluations of the elderly participating in the study. The aim of this report is, therefore, to describe the standardization process and reliability of anthropometric measurements carried out in the pilot study and during the final workshop, examining both intra- and inter-rater errors for measurements.

## Materials and Methods

### Population and Design

In December 2007, we conducted a theoretical session in Madrid (Spain) with the main researchers of the elderly EXERNET multi-centre study who planned to perform the anthropometric measurements. The aim of the workshop was to standardize the methodology and use it as a reference. From January to May 2008 pilot studies were conducted in the five Spanish cities (Zaragoza, Madrid, Toledo, León and Cáceres) and included a total of 98 seniors. These measurements were used to assess the intra-rater reliability of the anthropometric measures included in the study. In June 2008, a workshop was organized in the city of Toledo aiming to assess the inter-rater TEM, as well as the reliability of anthropometry measurements. Both measurements of intra-rater and inter-rater TEMs were carried out by the same anthropometrists (level 1 or with experience in the mentioned measurements through specific courses) who had been fully trained in the protocol by a reference anthropometrist (level 3), according to the methods of the International Society for the Advancement of Kinanthropometry (ISAK) [13]. In July 2008, we started the field work of the elderly EXERNET multi-centre study, which we finished in October 2009. The exclusion criteria for the pilot study and workshop were people under 65 years, cancer, dementia, dependent people and those who were living in nursing-homes. All applicable institutional and governmental regulations concerning the ethical issue of human volunteers were followed during this research. In brief, before the survey, all participants were informed by letter about the nature and purpose of the study. Written informed consent was obtained from all the subjects included. The study was performed according to the principles established with the Declaration of Helsinki (1964) as revised in 2000 in Edinburgh, and approved by the Clinical Research Ethics Committee of Aragón (18/2008).

### Anthropometric Methods

A portable stadiometer with 210 cm maximum capacity and a 0.1 cm precision (SECA 225, SECA, Hamburg, Germany) was used to measure height. Subjects stood with their scapula, buttocks and heels resting against a wall; the neck was held in a natural non-stretched position, the heels were touching each other, the toe tips formed a 45° angle and the head was held straight with the inferior orbital border in the same horizontal plane as the external auditive conduct (Frankfort’s plane) [14].

A portable bioelectrical impedance analyzer TANITA BC 418-MA (Tanita Corp., Tokyo, Japan) with a 200 kg maximum capacity and a ±100 g error margin was used to measure the body mass. Individuals removed shoes and heavy cloths prior to weighing.

Body-mass index (BMI) was estimated by dividing weight (kg) by height^2^ (m^2^). Both, body mass and BMI are included in this document as descriptive characteristics of the sample.

Waist and hip circumferences were measured in centimetres with a flexible non-elastic measuring tape (Rosscraft) to the nearest millimetre, according to the methods of the ISAK society [13]. Individuals were in a standing position with feet together and arms resting by their sides. Waist circumference (WC) was taken as the narrowest point between the inferior rib border and the iliac crest. The hip circumference (HC) measurement was taken at the point yielding the maximum circumference over the buttocks, with the tape held in a horizontal plane. Waist-to-hip ratio was calculated by dividing waist circumference (cm) by hip circumference (cm). This ratio is included in this document as a descriptive characteristic of the sample.

Knee height was measured in centimetres with a knee-height caliper (Rosscraft Surrey, British Columbia, Canada) to the nearest millimetre. This measurement was defined as the distance from the anterior surface of the thigh, just proximal to the patella, to the sole of the foot when the knee and ankle were flexed at a 90° angle [11].

### Intra-rater Study

In the pilot study 98 older adults (30 men, 68 women) were studied in the five cities. The main characteristics of these participants are shown in Table 1. Anthropometric measurements were carried out three times, but not consecutively; all the anthropometric variables were measured in order, and then the same measgturements were repeated two more times by the same observer.

**Table 1:** Characteristics of the groups studied in the intra-rater and inter-rater assessments.

	Intra-rater										Inter-rater	
	Zaragoza(*n* = 20)		Madrid(*n* = 20)		Toledo(*n* = 20)		León(*n* = 20)		Cáceres(*n* = 18)			
	Mean	SD	Mean	SD	Mean	SD	Mean	SD	Mean	SD	Mean	SD
Male/Female	12/8		3/17		10/10		3/17		2/16		6/4	
Age (y)	72.7	6.2	72.9	4.4	73.8	6.3	72.7	6.5	70.1	6.5	79.8	6.1
Body mass (kg)	71.0	12.6	69.3	11.1	67.3	10.3	67.0	11.3	67.9	10.6	66.6	17.1
Height (cm)	159.9	8.9	151.6	8.1	157.3	10.4	153.2	6.5	154.2	6.1	153.6	8.7
BMI (kg/m^2^)	27.7	4.1	30.1	4.0	27.2	3.6	28.5	4.1	28.5	3.5	27.8	4.6
Waist-to-hip ratio	1.0	0.1	1.0	0.1	0.9	0.2	0.9	0.1	0.9	0.1	0.9	0.1

Abbreviations: BMI, body mass index; SD, standard deviation.

### Inter-rater Study

Measurement of at least 10 subjects must be done for the calculation of intra- and inter-rater errors of measurement [7]. For inter-rater assessment, we studied 10 elderly living in the city of Toledo, who were different from those in the intra-rater sample. During the same morning, these persons were measured twice by each of the five observers. Each anthropometrist performed the complete set of anthropometric measurements. Participants consisted of six men and four women (79.1±6.1 years). The main characteristics of this sample are shown in Table 1.

### Statistical Analysis

The TEM is the most commonly used measure of precision. It was obtained by performing a number of repeated measurements on the same subject by the same observer (three measures by that observer) or two or more observers (one measure by five observers). The units of TEM were the same as those of the anthropometric measurement (centimeters). TEM was calculated with the widely used formula published elsewhere [7]. %TEM was also calculated using the following equation: [%TEM = (TEM/VAV)*100] where VAV is the variable average value. Results of %TEM for both intra- and inter-rater assessments are displayed in Table 2.

**Table 2:** Intra-rater and inter-rater TEM, %TEM and %*R* of anthropometric measurements.

	Intra-rater															Inter-rater		
	Zaragoza			Madrid			Toledo			León			Cáceres					
	TEM	%TEM	%*R*	TEM	%TEM	%*R*	TEM	%TEM	%*R*	TEM	%TEM	%*R*	TEM	%TEM	%*R*	TEM	%TEM	%*R*
Height (cm)	0.16	0.10	99.97	0.09	0.06	99.99	0.03	0.02	100	0.20	0.13	99.91	0.13	0.08	99.95	0.61	0.40	99.46
Knee height (cm)	0.24	0.48	99.53	0.17	0.37	99.73	0.07	0.15	99.95	0.50	1.02	96.70	0.43	0.91	99.84	0.75	1.55	94.65
Waist circumference (cm)	0.61	0.63	99.61	0.18	0.17	99.97	0.16	0.17	99.99	0.47	0.52	99.86	1.01	1.05	99.12	3.09	3.33	96.37
Hip circumference (cm)	0.50	0.50	99.49	0.21	0.20	99.92	0.41	0.37	99.98	0.57	0.54	99.46	0.96	0.88	98.58	2.70	2.61	91.31

Abbreviations: BMI, body mass index; SD, standard deviation, %*R*, percentage of coefficient of reliability; TEM, technical error of measurement.

Reliability (%R), which shows the proportion of the between-subject variance in a measured population that is free from measurement error, was calculated as previously described [7].

## Results

Table 2 shows the intra-rater TEM and %*R* for each anthropometric measurement in the five Spanish cities. For height, TEMs were smaller than 0.25 cm. For circumferences and knee height, TEMs were smaller than 1 cm, except for waist circumference in Cáceres (TEM = 1.01 cm). Reliability for heights and circumferences was always greater than 98% (except for knee height in León, %*R* = 96.70%).

Table 2 shows the inter-observer TEM and %*R* for each anthropometric measurement. TEMs were 0.61 cm for the height and 0.75 cm for the knee-height and ranged between 2.70 and 3.09 cm for the circumferences measured (hip and waist, respectively). Reliabilities for anthropometric measurements were always higher than 90%.

## Discussion

Coupled with the increased number of elderly people, an increase in the prevalence of overweight and obesity in this age group has occurred [9]. Overweight and obesity among elderly have a profound impact on health and mortality and therefore, they are a major public health concern [15].

One of the main objectives of the elderly EXERNET multi-centre study was to provide an updated prevalence of overweight and obesity in a representative sample of the non-institutionalized Spanish elderly population. To reach this aim, a great emphasis was put on the harmonization and standardization of measurements.

Due to anthropometry is less expensive and more practical than other techniques, it seems to be the most adequate method for epidemiological studies with a high number of subjects; therefore, reliability of body fat and fat distribution methods is extremely important to be defined [8]; however, to our knowledge, this is one of the first studies assessing the reliability of anthropometric measurements in this specific population. The most commonly used measures of precision are the TEM and *R* [7]. *R* indicates the proportion of between-subject variance in a measured population that is free from measurement error. Measures of *R* can be used to compare the relative reliability of different anthropometric measurements and of the same measurements in different age groups and to estimate sample size requirements in anthropometric surveys [8].

A generous allowance for measurement error might be up to 10% of the observed variance; this is equivalent to an *R* value of 90% or greater. Although this might be an acceptable lower limit, even at *R* values of approximately 95%, there is the occasional gross measurement error that is likely to have important consequences. Only when *R* is in the region of 99% is such an error unlikely. Even if *R* greater than 95% should be sought when possible [16], acceptable levels of measurement error are difficult to ascertain because TEM is related to the anthropometric characteristics of the group or population under investigation.

In the elderly EXERNETs pilot study, both the intra-rater and inter-rater TEM and %R values were above the required levels. Specifically, TEMs for heights and circumferences were frequently lower than 1 cm and %R greater than 98% (intra-rater), whereas inter-rater TEM and %R were more susceptible to error. Our results are similar than those found in other studies carried out in younger populations [8], [16]–[18]. Moreover, Ulijaszek and Kerr reviewed that there is a large variety of reliability among studies (ie. %R ranged from 86 to 99% for waist and 68 to 99% for hip circumference) [7], showing lower reliabilities than those found in our study.

The characteristic changes that occur during aging process in body composition (increase in fat mass and changes in tissue characteristics) could make more difficult the anthropometric assessment in this specific population, and therefore, the measurements could be more susceptible to error than in younger populations. Moreover, taking into account the average values of BMI and waist-to-hip ratio, the population samples were slightly overweight and then, TEMs of the anthropometric measurements (especially wait and hip circumferences) may be larger than those found in thinner populations. However, although TEM for waist and hip circumferences are quite large, our results suggest that low inter-rater errors can be achieved in elderly population. Therefore, this study is of great relevance because it is important to elucidate if similar reliabilities can be achieved in older populations compared with those found in adults or children [8], [16].

In our study, waist circumference was the anthropometric measurement with higher TEM in the inter-rater assessment. In addition to the fact that the population was slightly overweight, we think that the method used to measure this parameter could influence the results. Due to the narrowest point between the inferior rib border and the iliac crest use to be difficult to identify in elderly people, especially in those with an excess of abdominal fat, we suggest that WHO recommendations (midway between the inferior margin of the last rib and the crest of the ilium) could be better in this population group.

Knee height is a measure usually recorded in order to estimate by specific equations the height of individuals when is not possible to take this parameter through the normal procedure. However, due to standing height has been shown as an ideal technique for estimating the stature of individuals, including elderly people [19] and taking into account the high reliability of this parameter found in our study, it seems that stand height may be more appropriate and reliable than knee height to evaluate the size of seniors.

Anthropometric measurement error is unavoidable and should be minimized by paying close attention to every aspect of the data collection process. Regardless of the measurement made and the size of the error, it is better to know the size of error, as this will not only determine the confidence one has in the different measurements made, but will also influence the interpretation of anthropometric data collected.

These results allowed knowing the size of error in the different measurements made, which is of great importance for the interpretation of anthropometric data collected from different Spanish cities.

### Conclusion

The harmonization process, including the work shop and pilot study, guarantee the quality for the anthropometric measurements in the elderly EXERNET multi-centre study.
